# Comparative proteomics analysis of teleost intermuscular bones and ribs provides insight into their development

**DOI:** 10.1186/s12864-017-3530-z

**Published:** 2017-02-10

**Authors:** Chun-Hong Nie, Shi-Ming Wan, Tea Tomljanovic, Tomislav Treer, Chung-Der Hsiao, Wei-Min Wang, Ze-Xia Gao

**Affiliations:** 1 College of Fisheries, Key Lab of Agricultural Animal Genetics, Breeding and Reproduction of Ministry of Education/Key Lab of Freshwater Animal Breeding, Ministry of Agriculture, Huazhong Agricultural University, Wuhan, Hubei 430070 China; 2 Collaborative Innovation Center for Healthy Freshwater Aquaculture of Hubei Province, Wuhan, 430070 China; 3 Department for Fisheries, Beekeeping, Game management and Special Zoology, Faculty of Agriculture, University of Zagreb, Zagreb, Croatia; 4 Department of Bioscience Technology, Chung Yuan Christian University, Chung-Li, Taiwan; 5 Hubei Provincial Engineering Laboratory for Pond Aquaculture, Wuhan, 430070 Hubei China

**Keywords:** *Megalobrama amblycephala*, Intermuscular bones, Ribs, iTRAQ proteomics, MRM validation, Growth and differentiation

## Abstract

**Background:**

Intermuscular bones (IBs) and ribs both are a part of skeletal system in teleosts, but with different developing process. The chemical composition of fish IBs and ribs as well as the underlying mechanism about their development have not been investigated. In the present study, histological structures showed that one bone cavity containing osteoclasts were existed in ribs, but not in IBs of *Megalobrama amblycephala*. We constructed the first proteomics map for fish bones including IBs and ribs, and identified the differentially expressed proteins between IBs and ribs through iTRAQ LC-MS/MS proteomic analysis.

**Results:**

The proteins extracted from IBs and ribs at 1- to 2-year old *M. amblycephala* were quantified 2,342 proteins, with 1,451 proteins annotated with GO annotation in biological processes, molecular function and cellular component. A number of bone related proteins as well as pathways were identified in the study. A total of 93 and 154 differently expressed proteins were identified in comparison groups of 1-IB-vs-1-Rib and 2-IB-vs-2-Rib, which indicated the obvious differences of chemical composition between these two bone tissues. The two proteins (vitronectin b precursor and matrix metalloproteinase-2) related to osteoclasts differentiation were significantly up-regulated in ribs compared with IBs (*P* < 0.05), which was in accordance with the results from histological structures. In comparison groups of 1-IB-vs-2-IB and 1-Rib-vs-2-Rib, 33 and 51 differently expressed proteins were identified and the function annotation results showed that these proteins were involved in regulating bone development and differentiation. Subsequently, 11 and 13 candidate proteins in comparison group of 1-IB-vs-1-Rib and 1-IB-vs-2-IB related to bone development were validated by MRM assays.

**Conclusions:**

Our present study suggested the different key proteins involved in the composition of fish ribs and IBs as well as their growth development. These findings could provide important clues towards further understanding of fish skeletal system and the roles of proteins playing in regulating diverse biological processes in fish.

**Electronic supplementary material:**

The online version of this article (doi:10.1186/s12864-017-3530-z) contains supplementary material, which is available to authorized users.

## Background

Intermuscular bones (IBs) and ribs both are a part of skeletal system of fish. IBs, which occur only in teleosts amongst recent vertebrates, are segmental, serially homologous ossifications in the myosepta [1]. These small bones make big troubles for edible and surimi product processing, as well as reduce the flesh quality because they are difficult to remove [2]. The position in the myosepta distinguishes the IBs from ribs, which are found not in myosepta but in the muscle axialis [3, 4]. Meanwhile, ribs develop from cartilage bone, which develop from a mesenchymal cell population derive from the ventral somite. Unlike ribs, IBs develop directly from mesenchymal condensation. Since 1960s, researchers gradually began to realize the significance of studying IBs in fish [5–7]. However, these studies primarily focused on morphology, number and distribution of IBs in different fish species. As to the genetic resources of bone tissues for fish species, Vieira et al. used the comparative transcriptome analysis to characterize regulation mechanism of the vertebrae and gill arch in the gilthead sea bream (*Sparus auratus*) [8]. Our previous study has revealed the molecular properties of IBs through microRNA (miRNA) transcriptome analysis [9]. However, until now, the chemical composition of fish IBs and ribs has not been investigated. How many proteins of IBs and ribs may contain as well as which kind of differential expressed proteins existing between IBs and ribs remain unclear.

Proteomics is a large-scale, high-throughput, systematic study of a certain type of cell, tissue or body fluid composition of all proteins and an emerging discipline for proteins composition [10]. In post-genomic era, proteomics had been widely applied to various fields. Zhang et al. investigated the proteome of the oyster (*Crassostrea gigas*) shell by mass spectrometry (MS) [11]. Fan et al. applied 2-DE (two-dimensional electrophoresis) proteomics approach to investigate altered proteins in hepatopancreas of white shrimp (*Litopenaeus vannamei*) during cold stress [12]. As to fish species, Lucitt et al. constructed zebrafish (*Danio rerio*) embryonic protein expression profiles by 2DE LC-MS/MS (liquid chromatography-tandem mass spectrometry) method [13]. Kültz et al. studied biochemical differences of kidneys between marine and freshwater three spined sticklebacks (*Gasterosteus aculeatus*) by LC–MS/MS method [14]. Martyniuk et al. identified hepatic proteins associated with masculinization in female fathead minnow (*Pimephales promelas*) liver by using iTRAQ (isotope tags for relative and absolute quantitation) technology [15]. Those studies involved in diverse biological processes of proteomics in fish species, including embryonic development, masculinization process, environmental adaption, and so on. Multiple reaction monitoring (MRM) was a powerful tool for targeted proteomics and was an emerging field of proteomics, which had high reproducibility across complex samples [16, 17]. Currently, iTRAQ discovery combined with subsequent MRM confirmation has been adopted to determine key protein biomarkers in diseases. Muraoka et al. and Kaur et al. identified key proteins biomarkers in disease by iTRAQ proteomics combining with MRM assays, respectively [17, 18]. Liu et al. used iTRAQ proteomics combining with MRM assays to study the proteins associated with active immunization of subterranean termite (*Reticulitermes chinensis*) [19]. In the present study, the iTRAQ proteomics together with MRM analysis could contribute to a better understanding of the development mechanism for IBs and ribs in teleosts.

Blunt snout bream (*Megalobrama amblycephala* Yih, 1955), belonging to Cyprinidae, is a typical aquaculture species with IBs in China. This fish could reach the maturation at 2-year old and the growth of individuals decrease a lot once it gets the maturation. In the present study, we want to construct the first proteomics map for fish bones including IBs and ribs, as well as to identify the differentially expressed proteins between IBs and ribs. Moreover, we are also interested to find out which kinds of proteins play the important roles during the growing process of IBs and ribs. In order to obtain these objectives, iTRAQ technology and MRM assays were used for proteomics analysis of IBs and ribs from 1- to 2-year old *M. amblycephala*.

## Methods

All animals and experiments were conducted in accordance with the “Guidelines for Experimental Animals” of the Ministry of Science and Technology (Beijing, China). The study was approved by the Institutional Animal Care and Use Ethics Committee of Huazhong Agricultural University. All efforts were made to minimize suffering. All experimental procedures involving fish were approved by the institution animal care and use committee of the Huazhong Agricultural University.

### Sample collection

All experimental animals were collected from *M. amblycephala* selective population, which were bred in the Tuanfeng Fish Breeding Base of Huazhong Agricultural University. Six 1-year old and six 2-year old *M. amblycephala* individuals were selected. All specimens were collected on the same day and under the same conditions. The fish were euthanized immediately in well-aerated water containing the 100 mg/L concentration of tricaine methanesulfonate (MS-222) before tissue collection. Tissue samples including IBs and ribs were immediately collected, then snap-frozen in liquid nitrogen and stored at −80 °C.

### Morphological identification

Hematoxylin-Eosin (H-E) staining was used to observe the histological structures of IBs and ribs. Considering the convenient for the sectioning of bones, the encircled tissues were also collected along with the IBs and ribs. These tissues were fixed in Bouin’s liquid for 48 h, and embedded into paraffin blocks according to the routine procedures. Subsequently, the specimens were sectioned at 5 μm, and stained with hematoxylin and eosin.

### Protein preparation and SDS-PAGE electrophoresis

Equal amount of tissues samples (0.01 g IB and 0.02 g rib tissues) from each individual were mixed to grind into powder in liquid nitrogen and then the specific experimental method for protein extracted was referred from the method for protein preparation of Liu’s reference (Additional file 1: Text S1) [19]. The concentration of the protein was measured with Bradford method. Specific experimental operation for Bradford method can refer Toyama’s operation [20]. The proteins in the supernatant were kept at −80 °C for further analysis. IBs and ribs from three individuals were collected to extract protein for every stage and each stage possessed two biological replicates. So, we have eight protein samples, including 1-year old IB group (1-IB-I and 1-IB-II) and rib group (1-Rib-I and 1-Rib-II), 2-year old IB group (2-IB-I and 2-IB-II) and rib group (2-Rib-I and 2-Rib-II).

SDS-PAGE (sodium dodecyl sulfate – polyacrylamide gel electrophoresis) is the most common analytical technique used to separate and characterize proteins, which can detect the integrity of the protein samples [21, 22]. In this study, SDS-PAGE was used to distinguish the generally different biochemical compositions of IBs and ribs as well as identify the integrity of IBs and ribs’ protein samples. Aliquots containing 30 μg of each sample were separated on 10% resolving gels and SDS-PAGE analysis revealed clearly distinct band patterns for the IBs and ribs of *M. amblycephala* from the two developmental stages (Additional file 2: Figure S1). The samples were suitable for subsequent analysis and then subjected to trypsin digestion and LC–MS/MS analysis.

### Quantitative iTRAQ analysis

Total protein (100 μg) was taken out from each sample solution to perform the quantitative iTRAQ LC-MS/MS proteomic analysis. Details of iTRAQ labeling, strong cation exchange choematography (SCX) fractionation and LC-ESI-MS/MS analysis based on Triple TOF 5,600 during iTRAQ analysis are given in Additional file 1: Text S1.

The obtained raw data files were converted into MGF files using Proteome Discoverer 1.2 (PD 1.2, Thermo), 5,600 msconverter and the MGF file were searched. Proteins identification was performed by using Mascot 2.3.02 (Matrix Science, London, UK) against database containing 15,860 sequences. For protein identification, a mass tolerance of 20 Da (ppm) was permitted for intact peptide masses and 0.05 Da for fragmented ions, with allowance for one missed cleavages in the trypsin digests. Gln- > pyro-Glu (N-term Q), Oxidation (M), Deamidated (NQ) as the potential variable modifications, and Carbamidomethyl (C), iTRAQ8plex (N-term), iTRAQ8plex (K) as fixed modifications. The charge states of peptides were set to +2 and +3. Specifically, an automatic decoy database search was performed in Mascot by choosing the decoy checkbox in which a random sequence of database is generated and tested for raw spectra as well as the real database. To reduce the probability of false peptide identification, only peptides with significance scores (≥20) at the 99% confidence interval by a Mascot probability analysis greater than “identity” were counted as identified. Each confident protein identification involves at least one unique peptide.

Functional annotations of the proteins were conducted using Blast2GO program against the non-redundant protein database (NR, NCBI) and transcriptase data of intermuscular bones for 1-year old *M. amblycephala* which had been established by us previously (SRR1613326). The KEGG database (http://www.genome.jp/kegg/) and the COG (Clusters of Orthologous Groups of proteins) database (http://www.ncbi.nlm.nih.gov/COG/) were used to classify and group these identified proteins.

For protein quantization, it was required that a protein contains at least two unique peptides. The quantitative protein ratios were weighted and normalized by the median ratio in Mascot. Proteins with 2-fold change and p-value of statistical evaluation less than 0.05 in the comparison groups of 1-IB-vs-1-Rib (1-Rib as control), 2-IB-vs-2-Rib (2-Rib as control), 1-IB-vs-2-IB (2-IB as control) and 1-Rib-vs-2-Rib (2-Rib as control) were determined as differentially abundant proteins.

### MRM validation of differentially expressed proteins from iTRAQ

MRM assays were used to validate the differentially expressed proteins from iTRAQ analysis [19]. Details of the MRM analysis were referred to the Liu’s reference as well as described in Additional file 1: Text S1.

## Results

### Histological structures

**Fig. 1:**
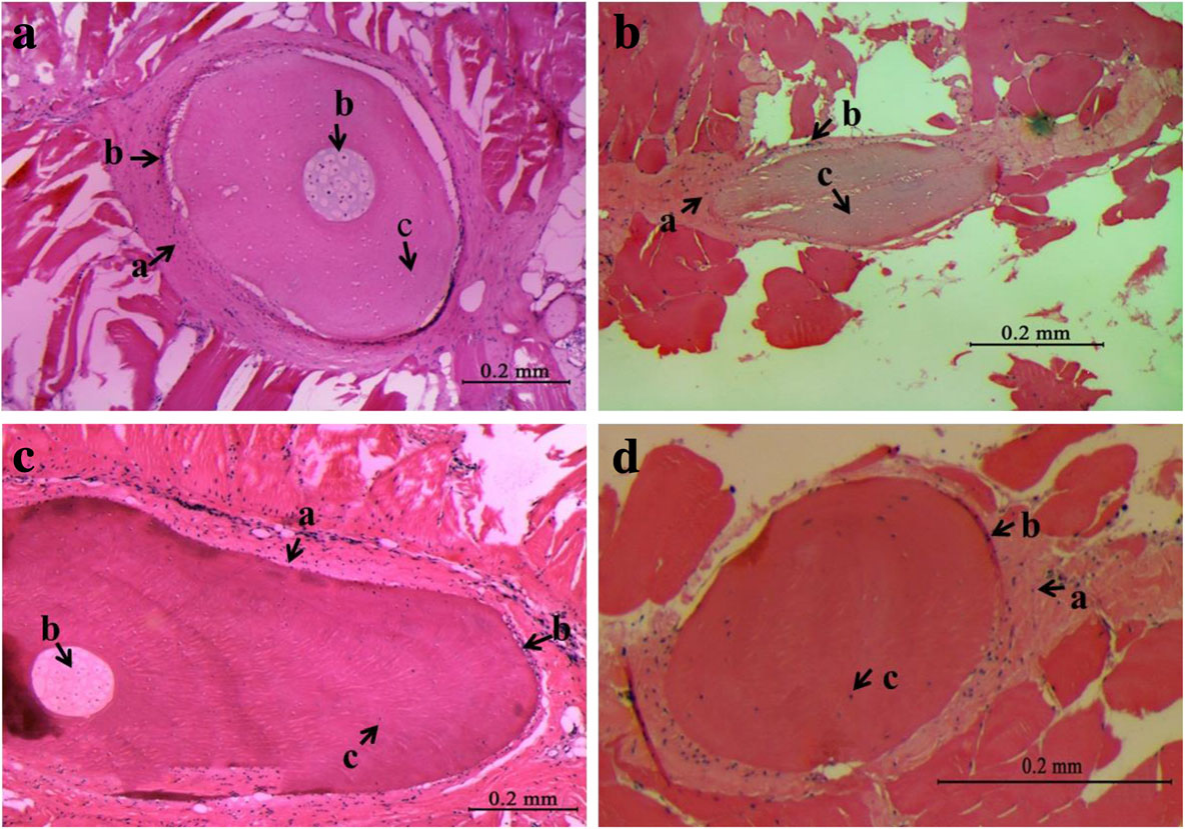
HE staining results for IBs and ribs of *M. amblycephala*, **a**-ribs of fish at 1-year old; **b**-IBs of fish at 1-year old; **c**-ribs of fish at 2-year old; **d**-IBs of fish at 2-year old. *a* showed osteoblast; *b* showed osteoclast; *c* showed osteocyte

To understand the histological characteristic of IBs and ribs, tissues were collected from 1- to 2-year old individuals of *M. amblycephala*. After stained by hematoxylin-eosin (H-E), their microstructures were observed and presented in Fig. 1. The three typical types of cells for bone formation, including osteoclasts, osteoblasts and osteocytes, were found in the histological structures of IBs and ribs. However, one bone cavity containing osteoclasts was identified in ribs from 1- to 2-year old individuals, but not in IBs.

### Proteome of IBs and ribs

In the present study, to improve the reliability of data, two biological replicates were included in the iTRAQ experiment for IBs and ribs at 1- and 2-year old *M. amblycephala*. After trypsinization and labeling with eight isobaric tags, the analytical separation and identification of the eight samples were performed. Totally 412,998 spectrums were generated, with 7,842 peptides and 2,342 proteins were identified (Additional file 3: Table S1; Additional file 2: Figure S2). In all identified proteins, 1,361 proteins were composed of only a single unique peptide and others were represented by two or more than two peptides. The repetitive analysis based on coefficient of variation (CV) between comparison groups was shown in Additional file 2: Figure S3, which indicated the good reproducibility of the data with the low mean CV values for all the comparison groups (0.11-0.13).

**Fig. 2:**
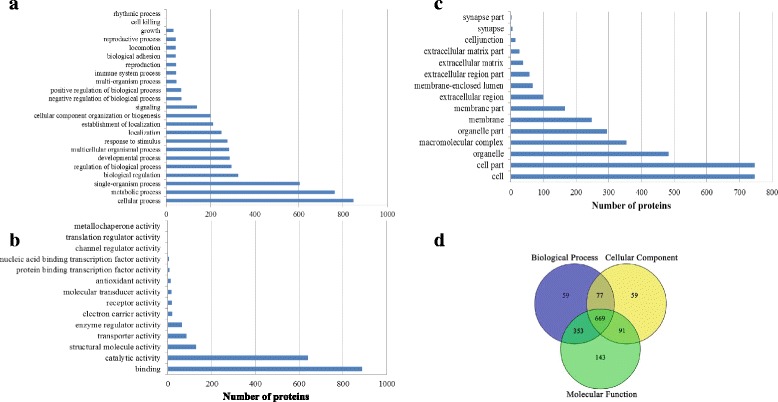
Proteins were functionally annotated for (**a**) biological process, (**b**) molecular function, as well as (**c**) cellular component and Venn diagram of proteins annotated for three processes (**d**)

A total of 1,451 proteins were functionally annotated with Gene Ontology (GO) (Additional file 3: Table S2)*.* There were 1,158 proteins played a role in biological processes with 59 unique proteins, with most represented proteins with GO terms of cellular process, single-organism process and metabolic process (Fig. 2a). There was also a wide range in the molecular functions with 1,256 proteins (including 143 unique proteins) being identified, with most represented proteins with GO terms of binding and catalytic regulator activity (Fig. 2b). The number of cellular components and unique cellular components represented by proteins was 896 and 59, respectively, with most proteins in GO terms of cell, cell part and organelle (Fig. 2c). A number of proteins were functioned in more than one GO terms (Fig. 2d).

Identified proteins analyzed with COGs were divided into 24 categories of function (Additional file 2: Figure S4), with most represented by proteins in R (general function prediction only), O (posttranslational modification, protein turnover, chaperones), J (translation, ribosomal structure and biogenesis) and Z (cytoskeleton). A total of 2,023 proteins (86.37%) were annotated with KEGG pathway and 235 pathways were identified (Additional file 3: Table S3). A large number of proteins were found to be involved in metabolic pathways, dilated cardiomyopathy (DCM), hypertrophic cardiomyopathy (HCM), focal adhesion, regulation of actin cytoskeleton as well as ECM-receptor interaction. There were 60, 41, 20, 17 and 11 proteins identified in the MAPK signaling, calcium signaling, TGF-β signaling, Wnt signaling as well as osteoclast differentiation pathways, respectively (Additional file 3: Table S4).

In all identified proteins, proteins associated with specific bone cell types including chondrocytes, osteoblasts, osteoclasts and osteocytes were identified, such as β-catenin and Col2a1a protein associated with chondrocytes, osteocalcin and annexin A1 related with osteoblasts, matrix metalloproteinase-2 and vitronectin b precursor correlated with osteoblasts, osteoglycin and parvalbumin-2 associated with osteocytes. Moreover, proteins associated with calcium or calcium-regulated and skeletal extracellular matrix were also identified, such as calmodulin and hemicentin-1 associated with calcium or calcium-regulated, matrilin-3a, type 1 collagen α1 and aggrecan core protein-like correlated with ECM. Detailed information for these proteins is listed in the Additional file 3: Table S5.

### Differentially expressed proteins between IBs and Ribs

**Fig. 3:**
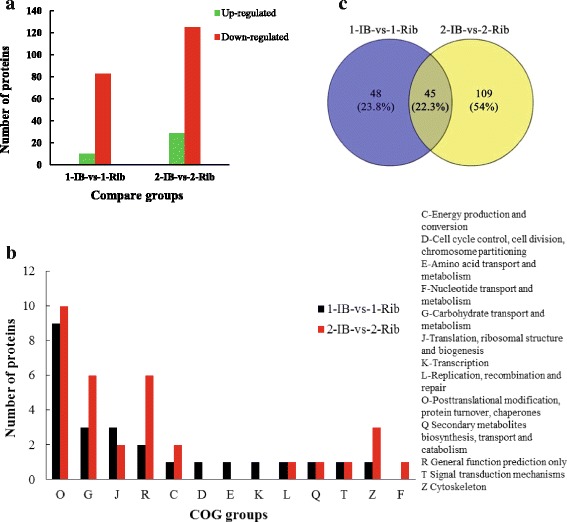
Comparison of proteins identified in 1-IB-vs-1-Rib and 2-IB-vs-2-Rib groups. **a** indicates differential expressed proteins, *X-axis*: names of the comparison groups; *Y-axis*: the number of differentially expressed proteins; *Red column*: up-regulation proteins; *Green column*: down-regulation proteins. **b** shows COG functional classification of differentially expressed proteins in two comparison groups. **c** shows Venn diagram for differentially expressed proteins between 1-IB-vs-1-Rib and 2-IB-vs-2-Rib

In the comparison groups of 1-IB-vs-1-Rib and 2-IB-vs-2-Rib, a total of 93 and 154 differentially expressed proteins (mean ratio > 2, *P* < 0.05) were identified by iTRAQ proteomics, including ten up-regulated and 83 down-regulated, 29 up-regulated and 125 down-regulated for each group, respectively (Fig. 3a; Additional file 3: Table S6-1; Table S6-2). In these two comparison groups, 20 and 37 proteins differentially expressed proteins were annotated by COG function classifications. It was found that the proteins for D (cell cycle control, cell division, chromosome partitioning) and E (amino acid transport and metabolism) function classifications were just presented in 1-IB-vs-1-Rib, not in 2-IB-vs-2-Rib; whereas the proteins for F (nucleotide transport and metabolism) function classification were just presented in 2-IB-vs-2-Rib, not in 1-IB-vs-1-Rib (Fig. 3b). In both comparison groups, the proteins for O (posttranslational modification, protein turnover, chaperones) functions classification accounted the largest proportion.

In GO annotation, the number of differentially expressed proteins made an effect on molecular function, biological process, and cellular component was 55, 40 and 36 in 1-IB-vs-1-Rib, while 72, 61 and 53 in 2-IB-vs-2-Rib, respectively. It was found that the amount of the proteins in hypertrophic cardiomyopathy (HCM) pathway were maximum. Noticeably, it was discovered that Ras homolog gene family, member Ac and thrombospondin-2 belonging to TGF-β signaling pathway were found in 2-IB-vs-2-Rib, and thrombospondin-1 as well as thrombospondin-2 belonging to TGF-β signaling pathway were found in 1-IB-vs-1-Rib.

Among the differentially expressed proteins, the two comparison groups shared 45 proteins, among which only one protein (laminin subunit α-2) was up-regulated and the rest of proteins were down-regulated (Additional file 3: Table S6-3). Among these proteins, Annexin A2a and osteocalcin being associated with osteoblast, parvalbumin isoform 1c being associated with osteocyte, were all down-regulation proteins. The proteins collagen α1(X) chain precursor, collagen α1(II) chain-like, matrilin 1 precursor biglycan and biglycan precursor associated with ECM were identified to be down-regulated. There were 48 and 109 proteins were unique differentially expressed in 1-IB-vs-1-Rib and 2-IB-vs-2-Rib groups, respectively (Fig. 3c; Additional file 3: Table S6-4; Table S6-5). For specific differentially expressed proteins in 1-IB-vs-1-Rib group, proteins associated with transport, metabolism of amino acid and nucleoside diphosphatase were dominant. Nine identified proteins were up-regulated including nidogen-2, tenascin-like, cartilage intermediate layer protein 1 precursor, actin related protein 2/3 complex, et al. Parvalbumin isoform 1d, parvalbumin1 and calcium-binding protein precursor associated with binding Ca^2+^ were found to be down-regulated. The down-regulated proteins of connective tissue growth factor precursor and insulin-like growth factor2 mRNA-binding protein3 were also identified. For specific differentially expressed proteins in 2-IB-vs-2-Rib, a total of 28 up-regulated proteins and 81 down-regulated proteins were identified. The mean ratio of myosin heavy chain fast skeletal type2 and troponin T3a was 2.69 and 2.5. Aggrecan core protein-like, matrilin-4 and matrilin-2 precursor associated with ECM were also found. Noticeably, the two proteins including vitronectin b precursor and matrix metalloproteinase-2, which are both related to osteoclast formation, had significantly higher expression in ribs compared to IBs (*P* < 0.05).

### Functional proteins during the growth of IBs and Ribs

**Fig. 4:**
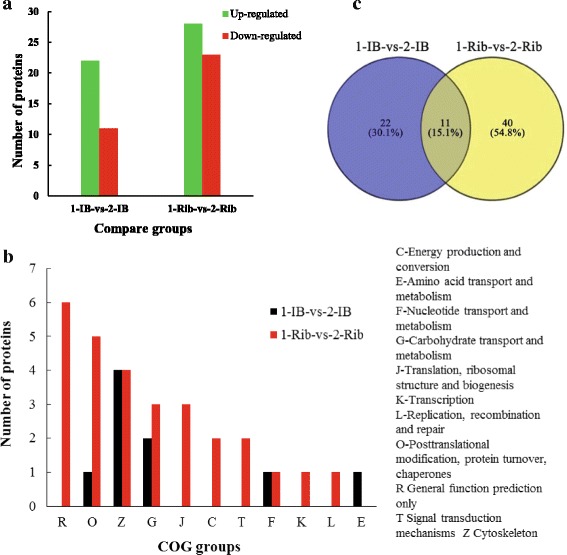
Comparison of proteins identified in 1-IB-vs-2-IB and 1-Rib-vs-2-Rib groups, **a** indicates differential expressed proteins, *X-axis*: names of the comparison groups; *Y-axis*: the number of differentially expressed proteins; *Red column*: up-regulation proteins; *Green column*: down-regulation proteins. **b** shows COG functional classification of differentially expressed proteins from iTRAQ data. **c** shows Venn diagram for 1-IB-vs-1-Rib and 2-IB-vs-2-Rib

A total of 33 and 51 differentially expressed proteins were identified by iTRAQ proteomics in 1-IB-vs-2-IB and 1-Rib-vs-2-Rib comparison groups (fold change > 2, *P* < 0.05), with 22 up-regulated and 11 down-regulated, 28 up-regulated and 23 down-regulated, respectively (Fig. 4a; Additional file 3: Table S7-1; Table S7-2). A total of 8 and 22 proteins were annotated in 1-IB-vs-2-IB and 1-Rib-vs-2-Rib by COG function classifications, respectively. There were just five classification functions in COG annotation identified for 1-IB-vs-2-IB differentially expressed proteins (Fig. 4b), including E (amino acid transport and metabolism), F (nucleotide transport and metabolism), G (carbohydrate transport and metabolism), O (posttranslational modification, protein turnover, chaperones) and Z (cytoskeleton), with the most number of proteins in Z function. Some classification functions was just identified for 1-Rib-vs-2-Rib group and the number of proteins for G (carbohydrate transport and metabolism) and O (posttranslational modification, protein turnover, chaperones) function classifications in 1-Rib-vs-2-Rib group was more than that in 1-IB-vs-2-IB group.

Among the differentially expressed proteins, the two comparison groups shared 11 proteins, among which nine proteins were up-regulated and two proteins were down-regulated (Additional file 3: Table S7-3). Parvalbumin1 and parvalbumin2 associated with calcium were found to be up-regulated. There were 22 specific differentially expressed proteins (13 up-regulated and nine down-regulated) in 1-IB-vs-2-IB group (Fig. 4c; Additional file 3: Table S7-4), among which titin, Fras1 related extracellular matrix 3 precursor, tnc protein as well as tenascin-like correlated with skeletal proteins were identified. Some proteins associated with myosin (myosin-7-like, myosin heavy chain fast skeletal type 2) and some proteins played a role in metabolism of amino acid permeases and energy (neutral α-glucosidase AB-like) were identified. Forty specific differentially expressed proteins (19 up-regulated and 21 down-regulated) were identified in 1-Rib-vs-2-Rib group (Additional file 3: Table S7-5). The value of ratio for actin-related protein 10 and myosin light chain was 5.84 and 5.55, respectively. Collagen α1(V) chain-like was also identified and its mean ratio was 2.71. Moreover, parvalbumin isoform 1d, parvalbumin isoform 4a and insulin-like growth factor 2 mRNA-binding protein3 were identified in 1-Rib-vs-2-Rib group. Some proteins correlated with Ca^2+^ metabolism and skeletal development were also identified.

**Fig. 5:**
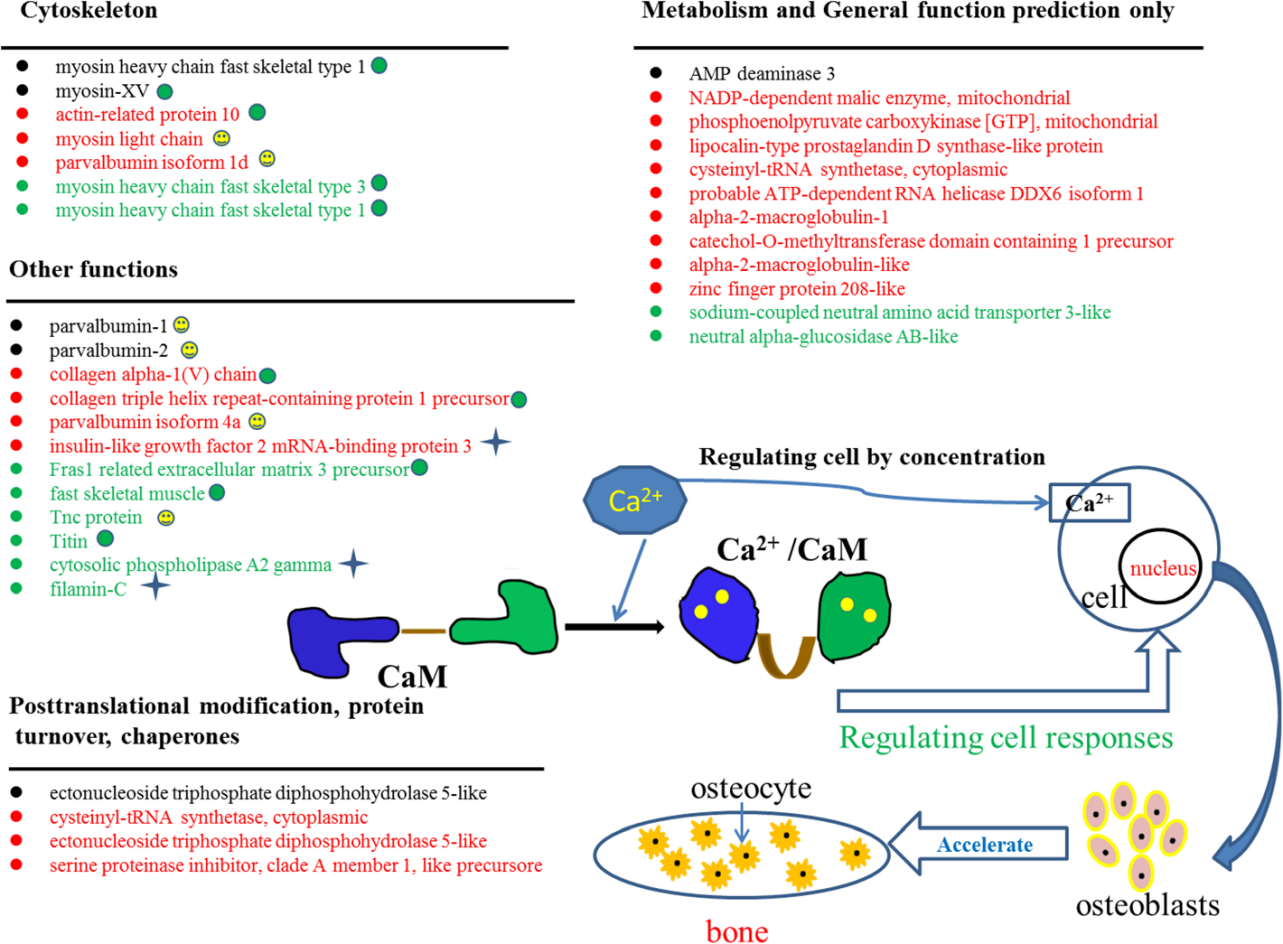
Distribution of the differentially expressed proteins in 1-IB-vs-2-IB and 1-Rib-vs-2-Rib by their functions. Using  marked proteins were associated with calcium or calcium-related process. Using  marked proteins related to bone formation and growth. Using  marked proteins related to osteoblast formation. The *black* type of proteins represented co-expressed differentially proteins in both 1-IB-vs-2-IB and 1-Rib-vs-2-Rib. The *red* type of proteins represented specific differentially expressed proteins in 1-Rib-vs-2-Rib. The *green* type of proteins represented specific differentially expressed proteins in 1-IB-vs-2-IB

Among the identified differential expressed proteins in the two comparison groups, there were many proteins associated with calcium or calcium-related, bone formation and growth (Fig. 5). In order to better understand how these proteins made an effect on bone development, the proteins annotated with COG function and associated with bone formation were selected to explain the possible mechanism for bone formation. Ca^2+^ may induce cell proliferation and growth by Ca^2+^ combining with calmodulin (CaM) and concentration of Ca^2+^ also made an effect on cell proliferation and growth. Some proteins, such as parvalbumin-1, tnc protein, myosin light chain, affect concentration of Ca^2+^ and combining of Ca^2+^ with CaM. Under the influence of protein-factor, cells can differentiate osteoblasts that can accelerate bone formation. The different expression levels of related proteins were indicated in Fig. 5, which could contribute to a better understanding of the basic information about which kind of proteins may play a more important role in the growth development of IBs and ribs.

**Fig. 6:**
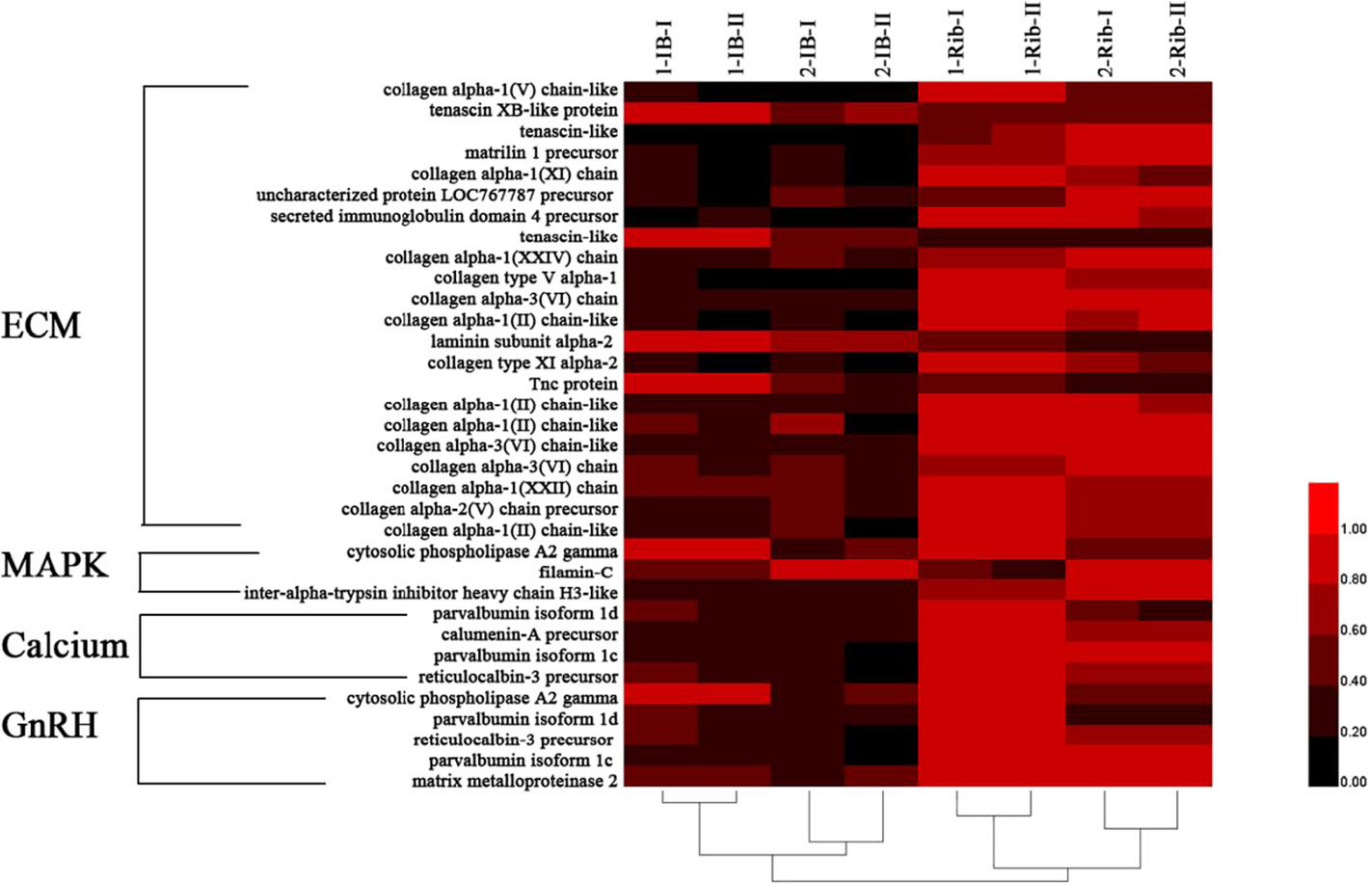
Cluster analysis of four pathway proteins in the 1-IB, 2-IB, 1-Rib and 2-Rib tissues. The color intensity indicates the level of protein expression. *Black* indicates a low level of protein expression or undetected protein; *red* indicates a high level of expression. 1-IB and 2-IB were IBs at 1 and 2 years old of *M. amblycephala*. 1-Rib and 2-Rib were ribs at 1 and 2 years old of *M. amblycephala*

From proteomics data, four pathways (ECM, MAPK, Calcium, GnRH pathway) proteins from eight samples were used to analyze the expression pattern based on their relative protein quantitation by cluster analysis (Fig. 6). In ECM pathway, 18 proteins (collagen α1(V) chain-like, matrilin 1 precursor, tnc protein, tenascin-like etc.) had most abundant expression in ribs tissue at one-1 old and four proteins (tenascin XB-like protein, tenascin-like, laminin subunit α-2, tnc protein) were relatively more abundant in IBs at 1-year old compared with other three tissues. Most of the proteins in calcium, MAPK and GnRH pathways exhibited high expression in rib tissues. All four pathway proteins exhibited lower expression in IBs at 2-year old excepted filamin-C.

### MRM validation for differential expressed proteins

**Fig. 7:**
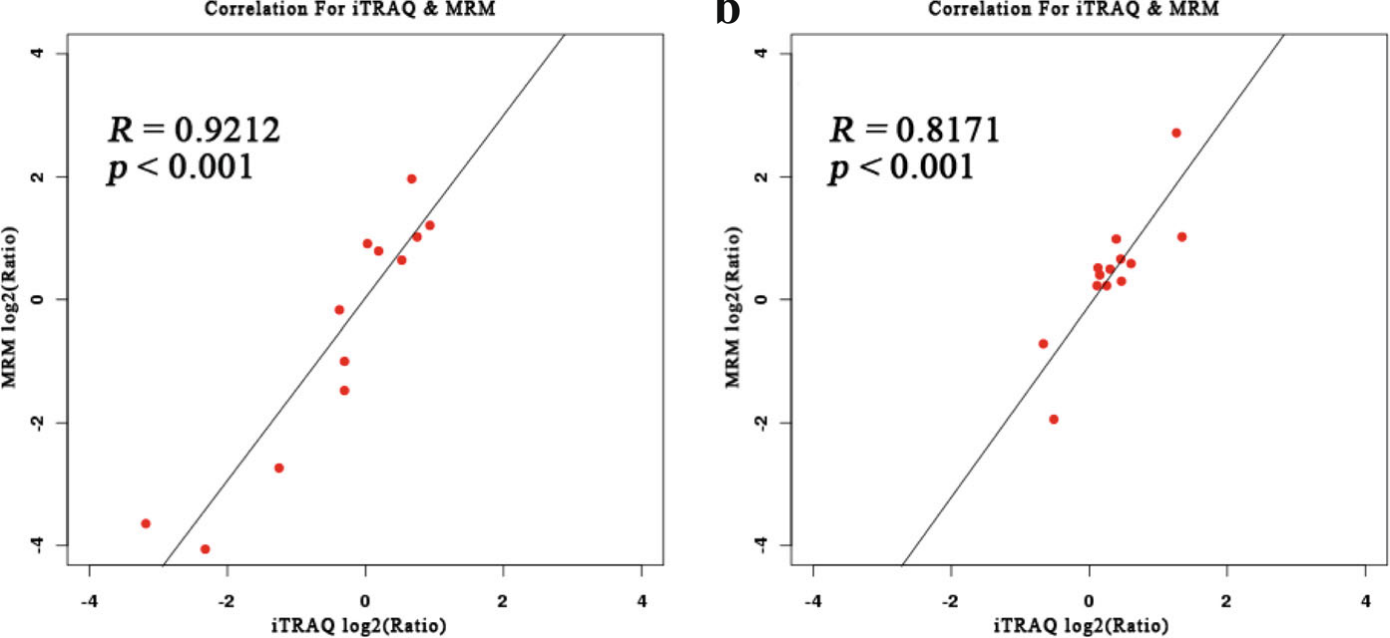
The correlation between iTRAQ quantified log_2_ (protein ratio) and MRM quantified log_2_ (protein ratio) for target proteins of the two comparison groups. **a** is for 1-IB-vs-1-Rib and **b** is for 1-IB-vs-2-IB

MRM analysis was a powerful tool that was used to validate targeted proteins. In the present study, MRM analysis succeeded in validating 11 and 13 proteins in comparison group of 1-IB-vs-1-Rib and 1-IB-vs-2-IB, respectively (Table 1). The transition information of two comparison groups of target proteins was showed in Additional file 3: Table S8. The log ratios of the quantitative data of these target proteins in two comparison groups from MRM were significantly positively correlated with those from iTRAQ (Fig. 7a, *R* = 0.9212, *p <* 0.001; Fig. 7b, *R* = 0.8171, *p <* 0.001).

**Table 1:** Proteins information from iTRAQ data and MRM validation in comparison groups of 1-IB-vs-1-Rib and 1-IB-vs-2-IB

Comparison	Protein	NCBI description	Mean ratio of iTRAQ	Mean ratio of MRM	*P*-value of MRM
1-IB-vs-1-Rib	CL1241.Contig2_All	transforming growth factor, beta-induced	1.44	1.56	0.09
	CL2864.Contig2_All	laminin, α-4	1.68	2.03	0.00
	CL2923.Contig2_All	osteoglycin precursor	1.02	1.88	0.02
	CL664.Contig4_All	fibronectin precursor	0.42	0.15	0.00
	Unigene109_All	collagen α2(XI)	0.20	0.06	0.02
	Unigene13384_All	Tnc protein	1.91	2.31	0.01
	Unigene16932_All	collagen α3(VI) chain	0.77	0.89	0.19
	Unigene19583_All	collagen α1(VI) chain	0.81	0.50	0.00
	Unigene441_All	osteomodulin	1.59	3.90	0.00
	Unigene7040_All	ictacalcin	0.11	0.08	0.00
	Unigene8331_All	decorin variant 1	1.14	1.73	0.00
1-IB-vs-2-IB	CL1241.Contig2_All	transforming growth factor, beta-induced	1.52	1.50	0.12
	CL2923.Contig2_All	osteoglycin precursor	1.37	1.58	0.06
	CL360.Contig2_All	myosin heavy chain fast skeletal type 1	0.63	0.61	0.09
	Unigene109_All	collagen type α2(XI)	1.09	1.43	0.74
	Unigene12601_All	myosin heavy chain fast skeletal type 3	2.40	6.56	0.05
	Unigene13384_All	Tnc protein	2.54	2.03	0.01
	Unigene16459_All	heat shock protein	1.11	1.32	0.17
	Unigene18499_All	collagen α6(VI) chain	1.08	1.17	0.59
	Unigene30256_All	collagen α1(XII) chain-like	1.38	1.23	0.34
	Unigene3121_All	calsequestrin 1a precursor	0.70	0.26	0.05
	Unigene441_All	osteomodulin	1.31	1.98	0.02
	Unigene7040_All	ictacalcin	1.19	1.17	0.82
	Unigene8331_All	decorin variant 1	1.23	1.41	0.00

In comparison group of 1-IB-vs-1-Rib, the six up-regulated proteins and six down-regulated proteins from iTRAQ data were consistent with the results from MRM analysis. Among them, the three significantly up-regulated proteins (laminin α-4; tnc protein; osteomodulin) and three significantly down-regulated proteins (fibronectin precursor; collagen α2(XI); ictacalcin) from iTRAQ data were also showed significantly up-regulated and down-regulated in MRM analysis (*p* < 0.05). In comparison group of 1-IB-vs-2-IB, the 11 up-regulated proteins and two down-regulated proteins from iTRAQ data have coincident expression with these from MRM analysis. Among them, the three significantly up-regulated proteins (tnc protein; osteomodulin; decorin variant 1) and one significantly down-regulated protein (calsequestrin 1a precursor) from iTRAQ data were also significantly up-regulated and down-regulated in MRM analysis.

## Discussion

The formation of bone includes complex multicellular activities involving various types of cells, with mainly cells being osteoclasts and osteoblasts [23]. Osteoblasts are related to structural bone and work in teams to build new bone [24]. Osteoclasts are the cells that degrade bone to initiate normal bone remodeling as well as conduct the function of bone resorption [25]. When osteoblasts become trapped in the matrix that they secrete, they become osteocytes. During the bone growth, bone cavity will be enlarged by osteoclasts through bone resorption process. Moreover, Ikeda et al. reported that osteoclasts activity was regulated with osteoblasts in certain extent [26]. Factors secreted from osteoclasts, such as ephrinB2, complement component 3a, collagen triple helix repeat containing 1, played a role in combining with osteoblasts receptor and osteoblasts differentiation, further affected bone mass and bone formation. In the present study, after HE staining for the IBs and the ribs, osteocyte is the most found cell in tissues, which is in accordance with human bone [27]. The osteoclasts and osteoblasts were both observed in histostructures of IBs and ribs, while bone cavity including osteoclasts was specially identified in ribs. These results indicated that there is less bone remodeling and bone resorption process in fish IBs compared to ribs, which maybe the reason that IBs cannot grow as big as ribs.

In this study, the first proteomics data were constructed for fish IBs and ribs in *M. amblycephala* and a total of 2,342 proteins were identified. There was a broad range in the biological functions represented by these proteins, with most represented by proteins included cellular process, metabolic process, single-organism process, biological regulation, developmental process, muleticellular organismal process. There has been great success in characterizing the proteome of fish tissues and the present study contributes to this growing body of literature. Lucitt et al. identified an impressive 1,384 proteins in zebrafish embryonic development stages at 72 hpf and 120 hpf, which revealed representation of protein classes relevant for cell function at both developmental stages, including proteins related to structure, transcription/translation, cell cycle, nucleotide metabolism, ion transport, carbohydrate, energy, and lipid metabolism [13]. De Souza et al. identified 5,716 proteins in zebrafish gill that represented acid–base regulation, stress, ion balance, and metabolism [28]. In the brain of zebrafish, Singh et al. identified more than 8,000 proteins representing hormones and hormone receptors [29]. In the female fathead minnows liver, Martyniuk et al. reported that 65% of the proteins had a biological role in metabolism and 42% had catalytic activity, which indicated that the female fathead minnows liver proteome is representative of the function of the liver in that it is the major tissue for detoxification and metabolism [14]. In this study, there were 36.81% proteins had a cellular role and metabolic process in biological process, 80.85% proteins played a role in binding and catalytic activity in molecular function, and 59.34% proteins made an effect on cell and membrane in cellular component in identified proteins of IBs and ribs, respectively, which suggested that proteins identified by this study may mainly made an effect on bone formation. Salmon et al. reported that 235 proteins were identified in human alveolar bone by LC-MS/MS, and most proteins involved in cellular process, biological regulation, and metabolic process [30]. Therefore, proteins function of IBs and ribs were similar with human alveolar bone in terms of GO annotation.

The identified proteins for IBs and ribs of *M. amblycephala* were categorized into 235 pathways, which were most represented by these proteins in metabolic, DCM, HCM pathways, focal adhesion, regulation of actin cytoskeleton as well as ECM-receptor interaction. Three pathways for focal adhesion, regulation of actin cytoskeleton as well as ECM-receptor interaction had certain correlation in bone development. Focal adhesion mainly affected cell proliferation. Actin is one of the important cytoskeleton proteins and made an effected on cell moving. ECM is comprised of basic structural proteins, collagens, proteoglycans, and glycoproteins [31]. ECM associated skeletal tissue of terrestrial vertebrates had been identified in vertebrae and gill arches of *S. auratus* [8]. In the present study, the different types of collagens were identified, such as collagen type II, collagen α1(XI) chain, collagen α1(X) chain precursor and so on. Proteins associated with TGF-β signaling pathway, Wnt signaling pathway and calcium signaling pathway were represented in IBs and ribs. Both of TGF-β signaling pathway and Wnt signaling pathway played an important role in the growth and differentiation of osteoblast, which was a part of skeletal tissue and involved in bone formation and differentiation [32, 33]. Proteins of calcium signaling pathway associated with Ca^2+^ or calcium-related including S100 calcium binding protein V2, hemicentin-1, calmodulin were identified. It had been reported that Ca^2+^ could be involved in many intracellular processes, including bone homeostasis, and calcium signaling is regulated by specific calcium-binding proteins [8]. Meantime, Concentration of Ca^2+^ could urge cell to occur stimulus response, and Ca^2+^ could directly act on osteoblast and affect bone formation [34–37]. So many bone related proteins identified in this study justifies the enrichment of the proteomics for fish bones.

Differentially expressed proteins of 1-IB-vs-1-Rib and 2-IB-vs-2-Rib were analyzed to identify the differentially expressed proteins between IBs and ribs. Osteocalcin and annexins A2a protein related with osteoblasts were identified as down-regulated proteins in both 1-IB-vs-1-Rib and 2-IB-vs-2-Rib groups, which mean they had higher expression in both 1- and 2-year old ribs compared with that of IBs. It had been reported that osteocalcin could induce bone regeneration and have distinct roles in the dentin mineralization process [38, 39], so it may play a critical role in the development and growth of ribs. Due to localization of annexins in areas of cartilage and bone mineralization, annexins made an important role in mineralization process. Kirsch reported that annexins A2, A5 and A6 played a role in physiological mineralization of skeletal tissues [40]. Annexins A2a may affect bone mineralization and make more effect on ribs than IBs. Moreover, biglycan, matrilin 1 precursor, collagen (collagen α1(V), collagen α2(XI)) were found as differentially expressed proteins in two comparison groups, and those proteins were a part of ECM pathway which was associated with bone mineralization. Above proteins associated with ECM, all was down-regulated proteins and played an important role in ribs growth compared with IBs development. Nidogen-2 protein was just identified as differentially expressed proteins in 1-IB-vs-1-Rib group with up-regulation level, not in 2-IB-vs-2-Rib group. Nidogen-2 was ubiquitous basement membrane proteins with a similar distribution in various organs during development, which could combine with cell membrane receptor and further promote bone formation [41]. The up-regulation of nidogen-2 proteins in 1-IB-vs-1-Rib group indicated it may play a critical role in IBs development compared with ribs. It was reported that vitronectin was characteristic markers for activated osteoclasts in mammals [42]. Mellis et al. reported that matrix metalloproteinase-9 could degrade the organic bone matrix [43]. The resorption of mineralized bone matrix by osteoclasts concurs with deposition of new bone by osteoblasts. Our results showed two related proteins (vitronectin b precursor and matrix metalloproteinase-2) were up-regulated in ribs compared with IBs, which was in accordance with the results from histological structures of ribs and IBs as there was no osteoclasts identified in IB internal tissues.

The differentially expressed proteins in 1-IB-vs-2-IB and1-Rib-vs-2-Rib groups were analyzed to identify the proteins for the growth development of IBs and ribs, respectively. Parvalbumin-1 and parvalbumin-2 associated with osteocyte were found being differentially expressed in both 1-IB-vs-2-IB and 1-Rib-vs-2-Rib groups. These two proteins had been reported to play a role in calcium signaling, through being correlated with calcium-binding protein and affecting relationship of troponin C combining with Ca^2+^ [44]. Therefore, the up-regulation of parvalbumin-1 and parvalbumin-2 in both groups indicated their important roles in the development of IBs and ribs. Tenascin-like, tnc, titin and fras1 related extracellular matrix 3 precursor were identified as specific differentially expressed proteins in 1-IB-vs-2-IB. Tenascin C (TNC) is an extracellular matrix glycoprotein synthesized by osteoblasts during bone growth and morphogenesis, which was reported to affect mineralization of osteoblast-like cells by matrix vesicles [45–47]. Powers et al. study had proved that titin protein made an effect on skeletal muscle sarcomeres [48]. According to our results, the up-regulation (Tenascin-like, fras1 related extracellular matrix 3 precursor, tnc, myosin heavy chain fast skeletal type 1) and down-regulation (titin, myosin heavy chain fast skeletal type 2) proteins may be the key proteins in regulating the IBs’ development. Collagen α1(V) chain-like and myosin light chain were identified as specific differentially expressed proteins for 1-Rib-vs-2-Rib. Collagen is the main composition of fish bone and connective tissue and involved in cell differentiation and multiplication [49]. There are about 27 different types of collagen having been identified in mammals, while just types I, II, V and XI had been reported in fish species [50]. Myosin light chain protein is correlated with Ca^2+^ binding protein and could affect bone formation. Lai et al. reported that myosin light chain affected contractility of skeletal muscle in female mice [51]. In this study, collagen α1(V) chain-like and myosin light chain both were up-regulated protein and they may play important roles in regulating ribs growth and development. DeMambro et al. reported that insulin-like growth factor-binding protein-2 is required for osteoclast differentiation and was related with the skeleton in mammals [52]. The up-regulation of insulin-like growth factor 2 mRNA-binding protein 3 identified in 1-Rib-vs-2-Rib may be similar with role of insulin-like growth factor-binding protein-2 and could regulate bone growth. These results suggest that there are different key proteins involved in development mechanisms of IBs and ribs.

In addition, we screened out four pathways proteins related to bone development and cluster analysis indicated their different expression levels in IBs and ribs. Calcium act as important second messengers for many intracellular processes including bone homeostasis [8]. Extracellular matrix (ECM), such as collagen, fibronectin, involved in bone mineralization and is critically important for cell growth, survival, differentiation and morphogenesis [53]. MAPK pathway is crucial for osteoclasts formation and differentiation and made an effect on bone mass [54]. GnRH can stimulate the expression of annexin A5 [55, 56], which had been reported to express in vertebrae of *S. auratus* and involved in bone mineralization [8, 40]. In the present study, different expression levels of the proteins from these four pathways indicated their possible important functions in the development of IBs and ribs.

In two comparison groups, MRM analysis was successfully used to validate differentially expressed proteins in each group. Among the validated proteins, collagens, fibronectin and osteomodulin are component of extracellular matrix and form tissue structure [53, 57]. Ohara et al. reported that fish collagen can promote the synthesis of proteoglycans in guinea pig tibial epiphysis [58], and therefore it may affect the treatment of osteoarthritis. Fibronectin and osteomodulin both were associated with collagen fibrils to contribute tissue strength [59, 60]. Tanaka et al. found that osteoglycin could affect osteoblasts type and made an effect on expression of runx2, and osterix [61]. In the present study, collagen, fibronectin precursor and osteomodulin as well as osteoglycin precursor from iTRAQ data were analyzed by MRM in the two comparison groups. In 1-IB-vs-1-Rib, the up-regulation of osteoglycin precursor and osteomodulin protein may play a more important role in IBs than ribs growth. Collagen α2(XI) and collagen α3(VI) chain and collagen α1(VI) chain were down-regulated proteins, suggesting that these three proteins may make a more effect on development of ribs than that of IBs. In 1-IB-vs-2-IB, the up-regulation of osteoglycin precursor, osteomodulin, collagen α2(XI) and collagen α3(VI) chain validated by MRM may be key proteins in IBs growth. In addition, laminin and tnc protein from iTRAQ date that were associated with Ca^2+^ were further validated by MRM, and these two proteins were up-regulated in two comparison groups may affect cell differentiation and further make an effect on bone formation.

Quantitative proteome is a powerful technology for large scare of differential proteins between different samples. Among the different methodologies, stable isotopes labeling by amino acids in cell culture (SILAC) [62], isotope-code affinity tags (ICAT) [63] as well as iTRAQ used in the present study are commonly employed. Although iTRAQ analysis allows identification of more proteins than previous 2-DE proteomics and more reliable quantification of the proteins, a large number of proteins can also not be detected by mass spectrometry. Therefore, the proportionally large number of proteins classified in this study is not necessarily an accurate representation of the protein composition related to fish IB and rib. Moreover, the performance of a concomitant proteomic and gene expression by throughput sequencing analyses would be expected to offer more reliable results.

## Conclusions

In conclusion, this study utilized iTRAQ methodology to construct the first proteomics map for fish bones including IBs and ribs, which increase knowledge about the proteins functioned in fish tissues. The identified 2,342 proteins represent the most comprehensive fish bone proteomes to date. Moreover, a total of 93 and 154 differentially expressed proteins were identified in comparison groups of 1-IB-vs-1-Rib and 2-IB-vs-2-Rib, as well as 33 and 51 differentially expressed proteins were identified in comparison groups of 1-IB-vs-2-IB and 1-Rib-vs-2-Rib. Some proteins were identified to have more important functions for the development of IBs or ribs, such as myosin-7-like, tnc protein, tenascin-like, fras1 related extracellular matrix3 precursor as well as myosin heavy chain fast skeletal type 3 for IBs, and actin-related protein 10, collagen α1(V) chain-like as well as parvalbumin isoform 1d for ribs. The obtained protein data from present study will contribute to a further understanding of the molecular mechanisms of IBs and ribs development and the roles of proteins playing in regulating diverse biological processes in fish.

## Additional files

Additional file 1:
**Text S1.** Detailed experimental protocols for iTRAQ analysis and MRM validation. (DOCX 22 kb)
Additional file 2: Figure S1. Separation of IBs and ribs of *M. amblycephala* from 1 to 2 year old by SDS-PAGE. **Figure S2.** The basic information statistics of proteome in this study. **Figure S3.** The repeatability analysis of data obtained from iTRAQ in different comparison groups based on CV (Coefficient of Variation) analysis. **Figure S4.** Functional classification of identified proteins. (DOCX 1365 kb)
Additional file 3: Table S1. Functional annotation information for the 2,342 identified proteins in IBs and Ribs of *M. amblycephala*. **Table S2.** Proteins information annotated with GO functions in IBs and Ribs of *M. amblycephala*. **Table S3.** KEGG pathway analysis of identified proteins in IBs and Ribs of *M. amblycephala*. **Table S4.** Detailed information for five pathways proteins of *M. amblycephala*. **Table S5.** Detailed information for proteins associated with bone cell of *M. amblycephala*. **Table S6.** Differential expressed proteins information in 1-IB-vs-1-Rib and 2-IB-vs-2-Rib. **Table S7.** Differential expressed proteins information in 1-IB-vs-1-IB and 1-Rib-vs-1-Rib. **Table S8.** The transition information of two comparison group of target proteins. (XLSX 20640 kb)

## References

[1] Patterson C, Johnson GD (1995). The intermuscular bones and ligaments of teleostean fishes.

[2] Ma LR (2012). The research progress on intermuscular bones of teleosts. Jiangsu Agric Sci.

[3] Hensley DA (1977). Larval development of Engyophrys senta (Bothidae), with comments on intermuscular bones in flatfishes. Bull Mar Sci.

[4] Johnson GD, Patterson C (2001). The intermuscular system of acanthomorph fish: a commentary. Am Mus Novit.

[5] Bing Z (1962). On the myoseptal spines of the carp (*Cyprinus carpio* L). Acta Zoolog Sin.

[6] Meng QW, Su JX, Li WD (1987). Comparative Anatomy of Fishes.

[7] Dong ZJ (2006). Preliminary study on intermuscular bones of several cultured cyprinids. J Shanghai Fisheries Univ.

[8] Vieira F (2013). Comparative analysis of a teleost skeleton transcriptome provides insight into its regulation. Gen Comp Endocrinol.

[9] Wan SM (2015). Identification of microRNA for intermuscular bone development in blunt snout bream (*Megalobrama amblycephala*). Int J Mol Sci.

[10] Yin W, Fu X, Li P (2014). Application research progress of proteomics. Biotech Bulletin.

[11] Zhang GF, Fang XD, Guo XM, Li L (2012). The oyster genome reveals stress adaptation and complexity of shell formation. Nature.

[12] Fan L (2016). Comparative proteomic identification of the hepatopancreas response to cold stress in white shrimp, *Litopenaeus vannamei*. Aquaculture.

[13] Lucitt MB (2008). Analysis of the zebrafish proteome during embryonic development. Mol Cell Proteomics.

[14] Martyniuk CJ, Alvarez S, Lo BP, Elphick JR, Marlatt VL (2012). Hepatic protein expression networks associated with masculinization in the female fathead minnow (*Pimephales promelas*). J Proteome Res.

[15] Kültz D (2015). Population-specific renal proteomes of marine and freshwater three-spined sticklebacks. J Proteomics.

[16] Addona TA (2009). Multi-site assessment of the precision and reproducibility of multiple reaction monitoring-based measurements of proteins in plasma. Nat Biotechnol.

[17] Muraoka S (2012). Strategy for SRM-based verification of biomarker candidates discovered by iTRAQ method in limited breast cancer tissue samples. J Proteome Res.

[18] Kaur P (2012). iTRAQ-based quantitative protein expression profiling and MRM verification of markers in type 2 diabetes. J Proteome Res.

[19] Liu L, Li GH, Sun PD, Lei CL, Huang QY (2015). Experimental verification and molecular basis of active immunization against fungal pathogens in termites. Sci Rep.

[20] Toyama H, Nishibayashi E, Saeki M, Adachi O, Matsushita K (2007). Factors required for the catalytic reaction of PqqC/D which produces pyrroloquinoline quinine. Biochem Biophys Res Commun.

[21] Lin FY (2014). Comparison of Several SDS-PAGE Gel Electrophoresis Staining Methods. J Anhui Agri Sci.

[22] Cao X (2016). iTRAQ-based comparative proteomic analysis of excretory–secretory proteins of schistosomula and adult worms of *Schistosoma japonicum*. J Proteomics.

[23] Parfitt AM (1994). Osteonal and hemi-osteonal remodeling: the spatial and temporal framework for signal traffic in adult human bone. J Cell Biochem.

[24] Wang H, Li YK (2011). Research on osteoblast differentiation regulatory factors. Int J Orthop.

[25] Boyce BF, Yao ZQ, Xing LP (2009). Osteoclasts have multiple roles in bone in addition to bone resorption. Crit Rev Eukaryot Gene Expr.

[26] Ikeda K, Takeshita S (2014). Factors and Mechanisms Involved in the Coupling from Bone Resorption to Formation: How Osteoclasts Talk to Osteoblasts. J Bone Metab.

[27] Tate MLK, Adamson JR, Tami AE, Bauer TW (2004). The osteocyte. Int J Biochem Cell Biol.

[28] De Souza AG, MacCormack TJ, Wang N, Li L, Goss GG (2009). Large-scale proteome profile of the zebrafish (*Danio rerio*) gill for physiological and biomarker discovery studies. Zebrafish.

[29] Singh SK, Rakesh KS, Ramamoorthy K, Saradhi AVP, Idris MM (2010). Proteome profile of zebrafish brain based on Gel LC-ESI MS/MS analysis. J Proteomics Bioinform.

[30] Salmon CR (2013). Proteomic analysis of human dental cementum and alveolar bone. J proteomics.

[31] Kielty CM, Grant ME (2003). The collagen family: structure, assembly, and organization in the extracellular matrix.

[32] Huang W, Yang SY, Shao JZ, Li YP (2007). Signaling and transcriptional regulation in osteoblast commitment and differentiation. Front Biosci.

[33] Xu L, Kong QQ (2014). Research Progress of key signaling pathways in osteoblast differentiation and bone formation regulation. Chinese J Reparative Reconstructive Surg.

[34] Guggino SE (1988). Phenylalkylamine-sensitive calcium channels in osteoblast-like csteosarcoma cells. J Biol Chem.

[35] Guggino SE, Lajeunesse D, Wagner JA, Snyder SH (1989). Bone remodeling signaled by a dihydropyridine-and phenaylalkylamine-sensitive calcium channel. Proc Natl Acad Sci.

[36] Gu GS (2002). Osteoblast calcium ion channels. Chin Rehab Clin Oncol.

[37] Wang MH (2011). Proteome profiles in medaka (*Oryzias melastigma*) liver and brain experimentally exposed to acute inorganic mercury. Aquat Toxicol.

[38] Liu C, Sun J (2015). Hydrolyzed tilapia fish collagen induces osteogenic differentiation of human periodontal ligament cells. Biomed Mater.

[39] Papagerakis P (2002). Investigation of osteocalcin, osteonectin, and dentin sialophosphoprotein in developing human teeth. Bone.

[40] Kirsch T (2005). Annexins - their role in cartilage mineralization. Front Biosci.

[41] Bechtel M (2012). Different domains in nidogen-1 and nidogen-2 drive basement membrane formation in skin organotypic cocultures. FASEB J.

[42] Proff P, Römer P (2009). The molecular mechanism behind bone remodeling: a review. Clin Oral Invest.

[43] Mellis DJ, Itzstein C, Helfrich MH, Crockett JC (2011). The skeleton: a multifunctional complex organ: the role of key signaling pathways in osteoclast differentiation and in bone resorption. J Endocrinol.

[44] Kültz D, Li J, Zhang X, Villarreal F, Pham T, Paguio D (2015). Population-specific plasma proteomes of marine and freshwater three-spined sticklebacks (*Gasterosteus aculeatus*). Proteomics.

[45] Ruth CE, Matthias C (2003). Tenascins: Regulation and putative functions during pathological stress. J Pathol.

[46] Sato R, Fukuoka H, Yokohama-Tamaki T, Kaku M, Shibata S (2016). Immunohistochemical localization of tenascin-C in rat periodontal ligament with reference to alveolar bone remodeling. Anat Sci Int.

[47] Li CZ (2016). Tenascin C affects mineralization of SaOS2osteoblast-like cells through matrix vesicles. Drug Discov Ther.

[48] Powers K, Nishikawa K, Joumaa V, Herzog W (2016). Decreased force enhancement in skeletal muscle sarcomeres with a deletion in titin. J Exp Biol.

[49] Lian XJ, Lu XX, Liu QS (2007). Research of fish collagen. Appl Res.

[50] Morvan-Dubois G, Le Guellec D, Garrone R, Zylberberg L, Bonnaud L (2003). Phylogenetic analysis of vertebrate fibrillar collagen locates the position of zebrafish α3(I) and suggests an evolutionary link between collagen α chains and Hox clusters. J Mol Evol.

[51] Lai SJ, Collins BC, Colson BA, Kararigas G, Lowe DA (2016). Estradiol modulates myosin regulatory light chain phosphorylation and contractility in skeletal muscle of female mice. Am J Physiol Endocrinol Metab.

[52] DeMambro VE (2012). Clemmons, Insulin-like growth factor-binding protein-2 is required for osteoclast differentiation. J Bone Miner Res.

[53] Rozario T, DeSimone DW (2010). The extracellular matrix in development and morphogenesis: a dynamic view. Dev Biol.

[54] Watts NB (2004). Relationship between changes in bone mineral density and vertebral fracture risk associated with risedronate: greater increases in bone mineral density do not relate to greater decreases in fracture risk. J Clin Densitom.

[55] Oikawa M, Dargan C, Ny T, Hsueh AJ (1990). Expression of gonadotropin-releasing hormone and prothymosin-alpha messenger ribonucleic acid in the ovary. Endocrinology.

[56] Kawaminami M, Shibata Y, Yaji A, Kurusu S, Hashimoto I (2003). Prolactin inhibits annexin5 expression and apoptosis in the corpus luteum of pseudopregnant rats: involvement of local gonadotropin-releasing hormone. Endocrinology.

[57] Mouw JK, Ou G, Weaver VM (2014). Extracellular matrix assembly: a multiscale deconstruction. Nat Rev Mol Cell Biol.

[58] Ohara H, Iida H, Ito K, Takeuchi Y, Nomura Y (2010). Effects of Pro-Hyp, a collagen hydrolysate-derived peptide, on hyaluronic acid synthesis using in vitro cultured synovium cells and oral ingestion of collagen hydrolysates in a guinea pig model of osteoarthritis. Biosci Biotechnol Biochem.

[59] Park ES (2009). Proteomics analysis of human dentin reveals distinct protein expression profiles. J Proteome Res.

[60] Tashima T, Nagatoishi S, Sagara H, Ohnuma S, Tsumoto K (2015). Osteomodulin regulates diameter and alters shape of collagen fibrils. Biochem Biophys Res Commun.

[61] Tanaka K (2012). Role of osteoglycin in the linkage between muscle and bone. J Biol Chem.

[62] Ong SE (2002). Stable isotope labeling by amino acids in cell culture, SILAC, as a simple and accurate approach to expression proteomics. Mol Cell Proteomics.

[63] Gygi SP (1999). Quantitative analysis of complex protein mixtures using isotope-coded affinity tags. Nat Biotechnol.

