# Supplementary material for: Comparative proteomics analysis of teleost intermuscular bones and ribs provides insight into their development
Source: BMC Genomics. 2017 Feb 10;18:147. doi: 10.1186/s12864-017-3530-z (PMC5301324; doi:10.1186/s12864-017-3530-z)
Supplement: Additional file 1: — Text S1. Detailed experimental protocols for iTRAQ analysis and MRM validation. (DOCX 22 kb) [file 12864_2017_3530_MOESM1_ESM.docx]

**Supplementary Information**

**Comparative proteomics analysis of teleost intermuscular bones and ribs provides insight into their development**

Chun-Hong Nie^1,2^, Shi-Ming wan^1,2^, Tea Tomljanovic^3^, Tomislav Treer^3^, Chung-Der Hsiao^4^, Wei-Min Wang^1^, Ze-Xia Gao^1, 2,*^

^1^*College of Fisheries, Key Lab of Agricultural Animal Genetics, Breeding and Reproduction of Ministry of Education/Key Lab of Freshwater Animal Breeding, Ministry of Agriculture, Huazhong Agricultural University, Wuhan, Hubei 430070, China*

^2^*Collaborative Innovation Center for Healthy Freshwater Aquaculture of Hubei Province, Wuhan 430070, China*

^3^*Department for Fisheries, Beekeeping, Game management and Special Zoology, Faculty of Agriculture, University of Zagreb, Zagreb, Croatia.*

^4^*Department of Bioscience Technology, Chung Yuan Christian University, Chung-Li, Taiwan*

*Corresponding author: Ze-Xia Gao, College of Fisheries, Huazhong Agricultural University, Wuhan, 430070 Hubei, China. E-mail address: [gaozexia@hotmail.com](mailto:gaozexia@hotmail.com)

**Experimental protocols**

**1. protein preparation**

Samples were ground into powder in liquid nitrogen, extracted with Lysis buffer (7 M Urea, 2 M Thiourea, 4% CHAPS, 40 mM Tris-HCl, pH 8.5) containing 1 mM PMSF and 2 mM EDTA (final concentration). After 5 min, 10 mM DTT (final concentration) was added to the samples. The suspension was sonicated at 200 W for 15 min and then centrifuged at 4 °C, 30 000 g for 15 min. The supernatant was mixed well with 5x volume of chilled acetone containing 10% (v/v) TCA and incubated at -20 °C overnight. After centrifugation at 4 °C, 30 000 g, the supernatant was discarded. The precipitate was washed with chilled acetone three times. The pellet was air-dried and dissolved in Lysis buffer (7 M urea, 2 M thiourea, 4% NP40, 20 mM Tris-HCl, pH 8.0-8.5). The suspension was sonicated at 200 W for 15 min and centrifuged at 4 °C, 30 000 g for 15 min. The supernatant was transferred to another tube. To reduce disulfide bonds in proteins of the supernatant, 10 mM DTT (final concentration) was added and incubated at 56°C for 1 h. Subsequently, 55 mM IAM (final concentration) was added to block the cysteines, incubated for 1 h in the darkroom. The supernatant was mixed well with 5x volume of chilled acetone for 2 h at -20°C to precipitate proteins. After centrifugation at 4°C, 30 000 g, the supernatant was discarded, and the pellet was air-dried for 5 min, dissolved in 500 μL 0.5 M TEAB (Applied Biosystems, Milan, Italy), and sonicated at 200 W for 15 min. Finally, samples were centrifuged at 4°C, 30 000 g for 15 min. The supernatant was transferred to a new tube and quantified using a Bradford kit (Bio-Rad). The proteins in the supernatant were kept at -80°C for further analysis

**2. SCX fractionation and LC-ESI-MSMS analysis based on Triple TOF 5600.**

SCX chromatography was performed with a LC-20AB HPLC Pump system (Shimadzu, Kyoto, Japan). The iTRAQ labeled peptide mixtures were reconstituted with 4 mL buffer A (25 mM NaH2PO4 in 25% ACN, pH 2.7) and loaded onto a 4.6×250 mm Ultremex SCX column containing 5-μm particles (Phenomenex). The peptides were eluted at a flow rate of 1mL/min with a gradient of buffer A for 10 minutes, 5-35% buffer B (25 mM NaH2PO4, 1 M KCl in 25% ACN, pH 2.7) for 11 min, 35-80% buffer B for 1 min. The system was then maintained at 80% buffer B for 3 min before equilibrating with buffer A for 10 min prior to the next injection. Elution was monitored by measuring the absorbance at 214 nm, and fractions were collected every 1 min. The eluted peptides are pooled as 20 fractions, desalted with a Strata X C18 column (Phenomenex) and vacuum-dried.

Each fraction was resuspended in buffer A (5% ACN, 0.1%FA) and centrifuged at 20000 g for 10 min, the final concentration of peptide was about 0.5 μg/μl on average. 10 μl supernatant was loaded on a LC-20AD nano HPLC (Shimadzu, Kyoto, Japan) by the auto sampler onto a 2 cm C18 trap column. Then, the peptides were eluted onto a 10 cm analytical C18 column (inner diameter 75) packed in-house. The samples were loaded at 8 μL/min for 4 min, then the 41 min gradient was run at 300 nL/min starting from 5 to 35% buffer B (95%ACN, 0.1%FA), followed by 5min linear gradient to 80%, and maintenance at 80% buffer B for 5 min, and finally return to 5% in 1 min.

**3. MRM validation of differentially expressed proteins from iTRAQ**

Samples were digested as described and spiked with 50 fmol of β-galactosidase for data normalization.  MRM analyses were performed on a QTRAP 5500 mass spectrometer (SCIEX, Framingham, MA, USA) equipped with LC-20AD nanoHPLC system (Shimadzu, Kyoto, Japan). The Mobile phase consisted of solvent A, 0.1% aqueous formic acid and solvent B, 98% acetonitrile with 0.1% formic acid. Peptides were separated on a C18 column (0.075 x 150 mm column, 3.6 μm) at 300 nL/min, and eluted with a gradient of 5%-30% solvent B for 38 min, 30%-80% solvent B for 4 min, and maintenance at 80% for 8 min. For the QTRAP 5500 mass spectrometer, spray voltage of 2400 V, nebulizer gas of 23 p.s.i., and a dwell time of 10 ms were used. Multiple MRM transitions were monitored using unit resolution in both Q1 and Q3 quadrupoles to maximize specificity.

The obtained data was used Skyline software to integrate the raw file generated by QTRAP 5500 (SCIEX, Framingham, MA, USA) and used an iRT strategy to define a chromotography of a given peptide agianst a spectral library. We use MSstats with the linear mixed-effects model The P values were adjusted to control the FDR at a cutoff of 0.05. All proteins with a P value below 0.05 and a fold change larger than 1.5 are considered significant.
