# Supplementary material for: Comparative proteomics analysis of teleost intermuscular bones and ribs provides insight into their development
Source: BMC Genomics. 2017 Feb 10;18:147. doi: 10.1186/s12864-017-3530-z (PMC5301324; doi:10.1186/s12864-017-3530-z)
Supplement: Additional file 2: Figure S1. — Separation of IBs and ribs of M. amblycephala from 1 to 2 year old by SDS-PAGE. Figure S2. The basic information statistics of proteome in this study. Figure S3. The repeatability analysis of data obtained from iTRAQ in different comparison groups based on CV (Coefficient of Variation) analysis. Figure S4. Functional classification of identified proteins. (DOCX 1365 kb) [file 12864_2017_3530_MOESM2_ESM.docx]

**Supplementary Figures**

**Comparative proteomics analysis of teleost intermuscular bones and ribs provides insight into their development**

Chun-Hong Nie^1,2^, Shi-Ming wan^1,2^, Tea Tomljanovic^3^, Tomislav Treer^3^, Chung-Der Hsiao^4^, Wei-Min Wang^1^, Ze-Xia Gao^1, 2,*^

^1^*College of Fisheries, Key Lab of Agricultural Animal Genetics, Breeding and Reproduction of Ministry of Education/Key Lab of Freshwater Animal Breeding, Ministry of Agriculture, Huazhong Agricultural University, Wuhan, Hubei 430070, China*

^2^*Collaborative Innovation Center for Healthy Freshwater Aquaculture of Hubei Province, Wuhan 430070, China*

^3^*Department for Fisheries, Beekeeping, Game management and Special Zoology, Faculty of Agriculture, University of Zagreb, Zagreb, Croatia.*

^4^*Department of Bioscience Technology, Chung Yuan Christian University, Chung-Li, Taiwan*

*Corresponding author: Ze-Xia Gao, College of Fisheries, Huazhong Agricultural University, Wuhan, 430070 Hubei, China. E-mail address: [gaozexia@hotmail.com](mailto:gaozexia@hotmail.com)

**
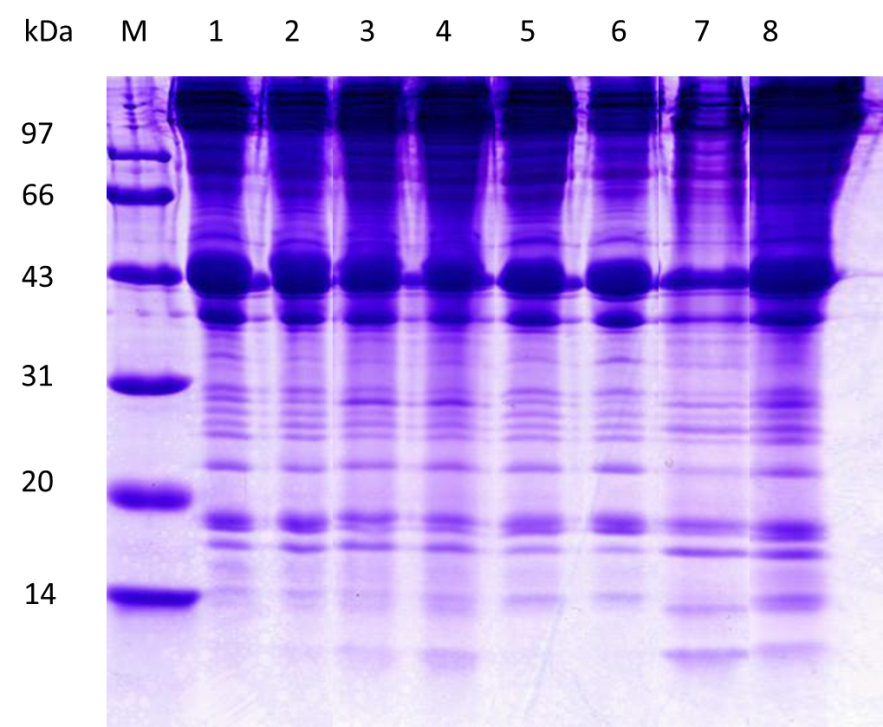
**

M, marker

1, 1-IB-I

2, 1-IB-II

3, 1-Rib-I

4, 1-Rib-II

5, 2-IB-I

6, 2-IB-II

7, 2-Rib-I

8, 2-Rib-II

**Figure S1**. Separation of IBs and ribs of *M. amblycephala* from one and two year old by SDS-PAGE.

**
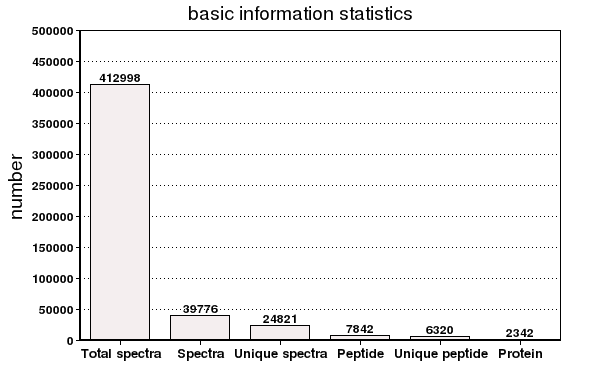
**

**Figure S2.** The basic information statistics of proteome in this study.

**
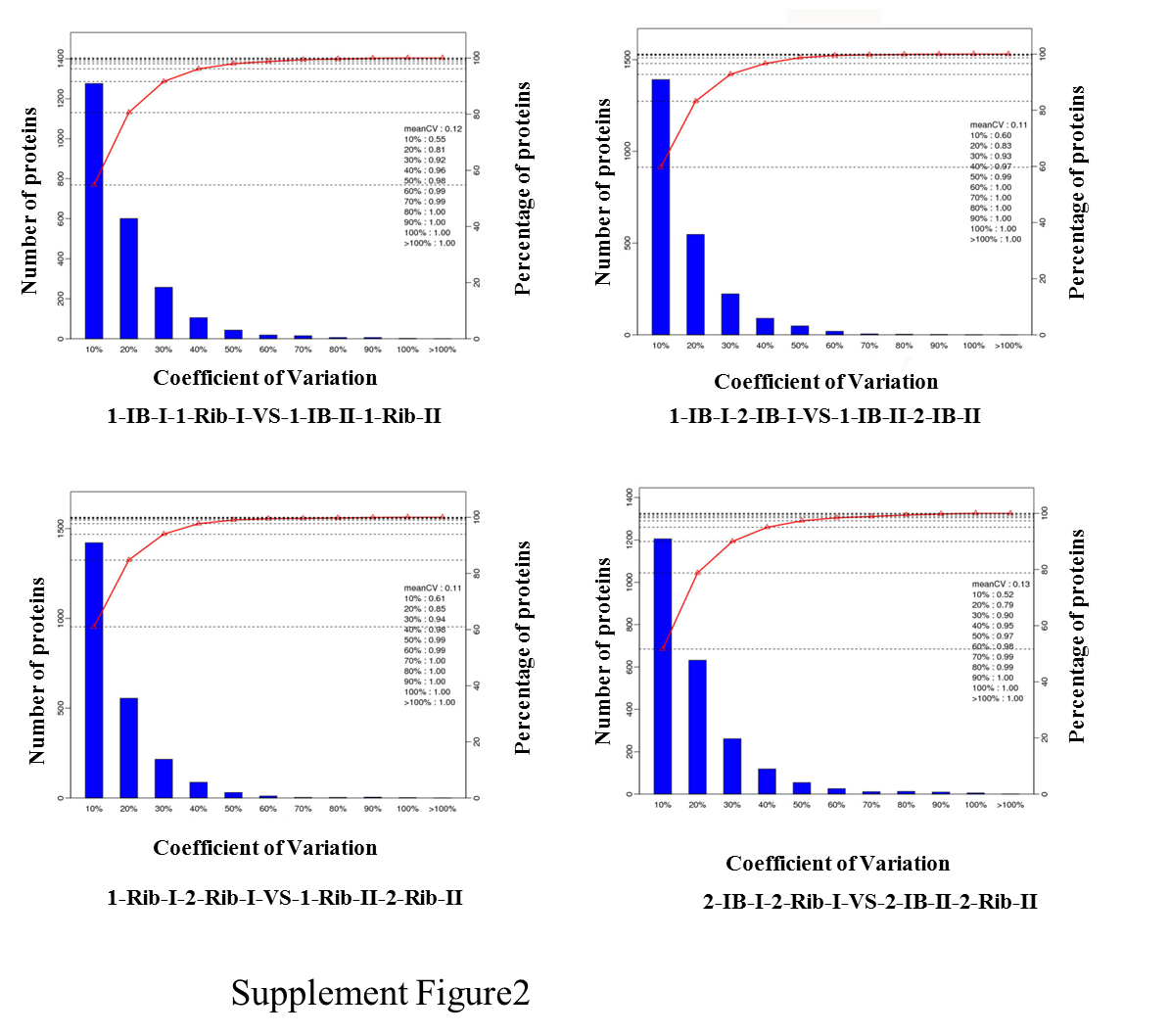
**

**Figure S3**. The repeatability analysis of data obtained from iTRAQ in different comparison groups based on CV (Coefficient of Variation) analysis.

COG groups

**Figure S4.** Functional classification of identified proteins. A, RNA processing and modification; B, Chromatin structure and dynamics; C, Energy production and conversion; D, Cell division and chromosome partitioning; E, Amino acid transport and metabolism; F, Nucleotide transport and metabolism; G, Carbohydrate transport and metabolism; H, Coenzyme metabolism; I, Lipid metabolism; J, Translation, ribosomal structure and biogenesis; K, Transcription; L, DNA replication, recombination, and repair; M, Cell envelope biogenesis, outer membrane; N, Cell motility and secretion; O, Posttranslational modification, protein turnover, chaperones; P, Inorganic ion transport and metabolism; Q, Secondary metabolite biosynthesis, transport, and catabolism; R, General function prediction only; S, Function unknown; T, Signal transduction mechanisms; U, Intracellular trafficking and secretion; V, Defense mechanisms; Y, Nuclear structure; Z, Cytoskeleton.
